# O047. The sound-induced flash illusions reveal visual cortex hyperexcitability in cluster headache

**DOI:** 10.1186/1129-2377-16-S1-A92

**Published:** 2015-09-28

**Authors:** Giuseppe Cosentino, Simona Talamanca, Maria Aprile, Simona Maccora, Roberta Baschi, Laura Pilati, Salvatore Di Marco, Brigida Fierro, Filippo Brighina

**Affiliations:** Dipartimento di Biomedicina Sperimentale e Neuroscienze Cliniche (BioNeC), Università di Palermo, Palermo, Italy

## Objectives

Pathophysiology of cluster headache (CH) is not well-known. Although posterior hypothalamus has been suggested to play a pivotal role, evidence exists of a more diffuse involvement of the central nervous system including brainstem and cerebral cortex. In this regard, we recently observed increased motor cortical excitability in episodic CH patients both outside and inside bout[1].

The sound-induced flash illusions (SIFI) represent an example of multisensory integration, and provide a tool to indirectly explore the excitability state of the visual cortex[2]. SIFI are classified as “fission” and “fusion” illusions. When one visual stimulus (flash) is accompanied by two or more auditory stimuli (beeps), it is often perceived as multiple flashes (fission illusion). Conversely, fusion illusion occurs when subjects perceive less number of flashes when these are presented with only one beep. On such bases, here we used SIFI to explore excitability of visual cortex in CH patients.

## Materials and methods

SIFI were examined in ten untreated patients with episodic CH and in twelve age- and sex-matched healthy volunteers. Five out of the ten patients were evaluated both inside (in the interval between two pain attacks) and outside bout. Visual stimuli were accompanied by beeps in different combinations to evaluate both fission illusion (one flash presented with a number of beeps varying between 0 and 4) and fusion illusion (2-4 flashes accompanied by only one beep).

## Results

The fission but not the fusion illusion was significantly reduced in CH patients with respect to healthy controls. No significant differences were observed between bout and outside bout phases in patients evaluated both in the ictal and interictal state.

## Discussion

The present results provide evidence of increased visual cortical excitability in CH that is detectable not only during the bout, but also in the pain-free period. This is in agreement with previous findings by our group of increased motor cortex excitability in CH inside and outside bout.

## Conclusions

These results strengthen the notion that an abnormal cortical excitability state exists in CH. Moreover, findings in CH patients are very similar to those observed in migraine with aura patients[3], thus supporting the idea that CH and migraine could share at least some common pathophysiological pathways.

On such bases, we suppose that visual cortex hyperexcitability could play a pathogenic role in CH as well as in migraine with aura. This suggestion may also be supported by the rather frequent occurrence of aura symptoms in CH sufferers.

Written informed consent to publication was obtained from the patient(s).
